# Supplementary material for: Application of Polymethylpentene, an Oxygen Permeable Thermoplastic, for Long-Term on-a-Chip Cell Culture and Organ-on-a-Chip Devices
Source: Micromachines (Basel). 2023 Feb 24;14(3):532. doi: 10.3390/mi14030532 (PMC10053898; doi:10.3390/mi14030532)
Supplement: Supplementary file 1 [file micromachines-14-00532-s001.zip › micromachines-2201590-supplementary.pdf]

SUPPLEMENTARY INFORMATION TO

Application of polymethylpentene, an oxygen permeable thermoplastic, for long-term on-a-chip cell culture and organ-on-a-chip devices

S1 Measurement of O_2 and CO_2 permeability of polymers

Permeation measurements were conducted using the constant-pressure method, analogue to ASTM D3985 - 17, though employing gas chromatography (GC) analysis on the permeate stream, in a home-made permeation set-up at SINTEF Industry, Norway (figure S1a). Samples are placed in a home-made circular permeation cell (figure S1b) in which a porous stainless plate is applied to support the sample during higher pressure conditions. Sealing is easily obtained by clamping the sample between the O-rings installed on the front and back-side of the module.

The permeation cell is placed in a Memmert UF450 forced air circulation oven for temperature control. Automated mass flow controllers (MFC) (Bronkhorst High-Tech, F-201CV) are used to control the gas supply to the front and back side of the module. The pressure of the front side is controlled with the help of a back-pressure controller (Bronkhorst High-Tech, P-702CV, max. 30 bars). The permeate side is always at atmospheric pressure, and the permeate flow is measured using an automated mass flow meter (Bronkhorst High-Tech, F-101D, size 100 mL/min). In order to determine the amount of permeated gas, a two-channel (MS and PPU column) μ-GC (Agilent 490) equipped with thermal conductivity detectors (TCD) is employed to monitor the concentration of the permeated components, in this case O_2 and CO_2, in the Ar sweep gas.

The permeation experiments for subsequently O_2 and CO_2 are performed as follows for the PMP and PDMS films:

1. Seal sample in cell, and connect to set-up;
2. Expose the cell to a small overpressure of N_2 (1 barg) employing an Ar sweep flow rate (~20 Nml/min) on the other side of the sample (typically ~2 hours); Temperature is left to stabilize to 37°C;
3. At stable condition of temperature and pressure, and GC analysis, N_2 is replaced by O_2;
4. O_2 gradually permeates through the sample, and the breakthrough is determined by the GC. The permeation is left to stabilize to obtain the permeate rate at 37°C (until the next day);
5. Subsequently, the permeation is as well verified at 0 and 0.5 barg after which the O_2 is replaced by CO_2 and the tests are repeated.

For polycarbonate, the procedure was the same, except an overpressure of 4 barg was utilized.

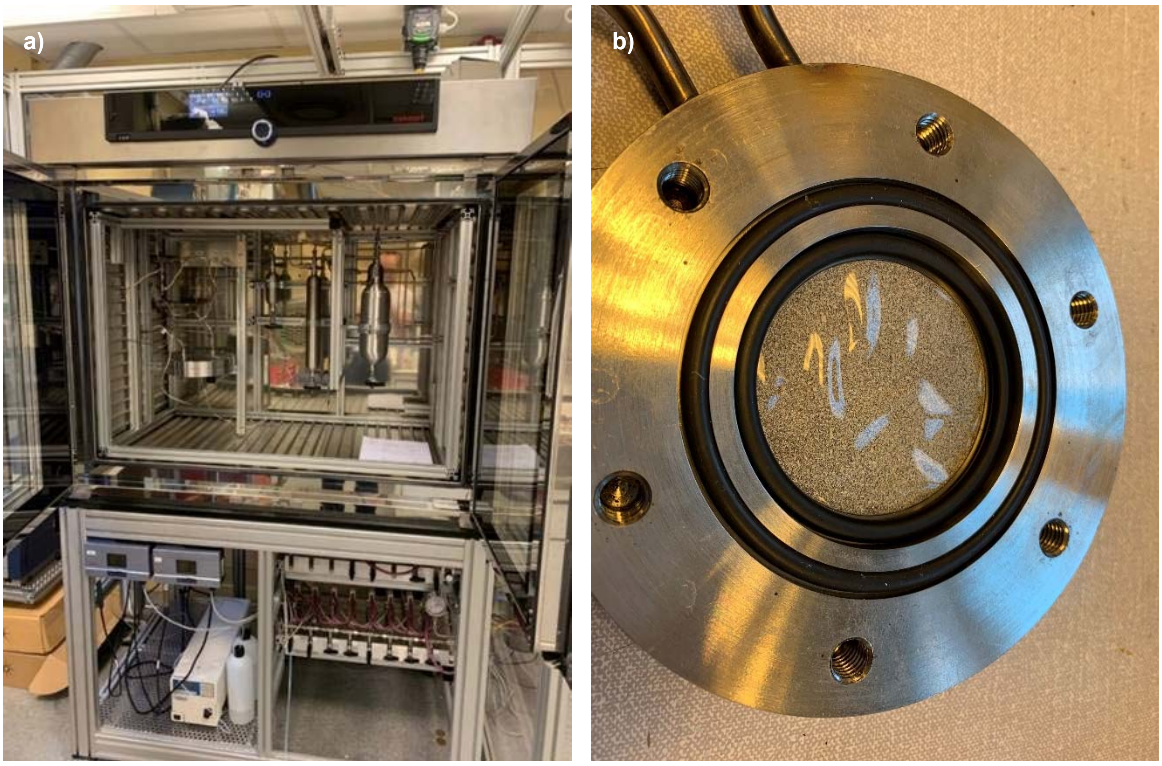

Figure S1: Set-up A picture of (a) the home-made permeation set-up and (b) the home-made circular permeation cell with the PDMS film after the gas permeation test (right).

S2 Transmission light imaging of adherent cells on commercial PMP film without polishing

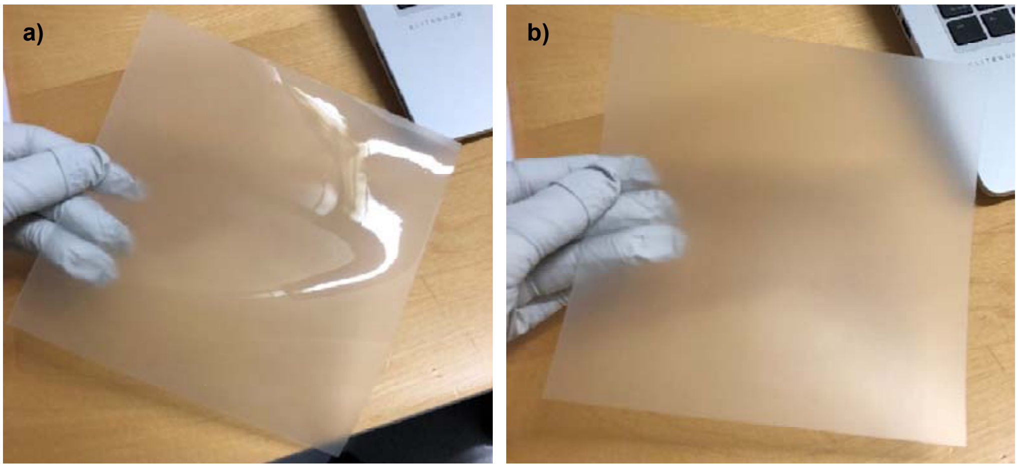

Figure S2: Commercial PMP film The commercial PMP film from Goodfellow has (a) one gloss and (b) one matt side, although it is stated from the manufacturer to be "Clear/transparent".

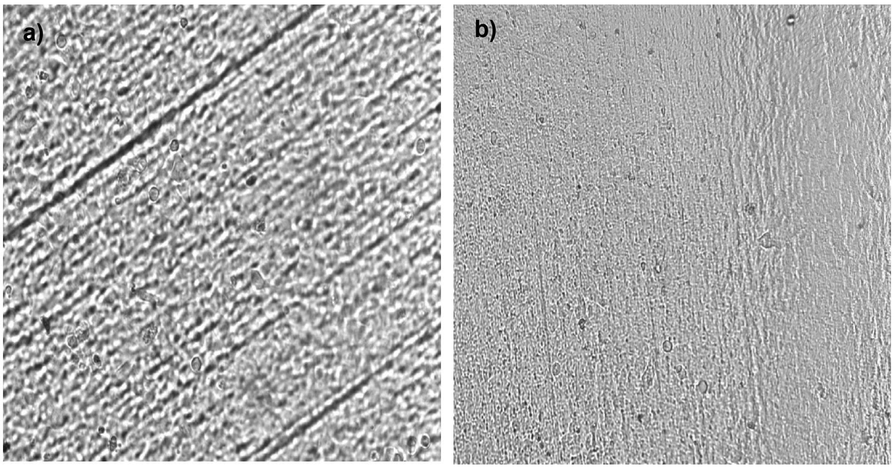

Figure S3: Transmission light images of cells grown on unpolished PMP film Transmission light images of adherent cells growing (a) on the gloss side and (b) on the matt side of the commercial PMP film in its native, unpolished state. On this unpolished PMP, it is not possible to observe the cells' morphology and state.

S3 Raw data from monitoring of oxygen concentration

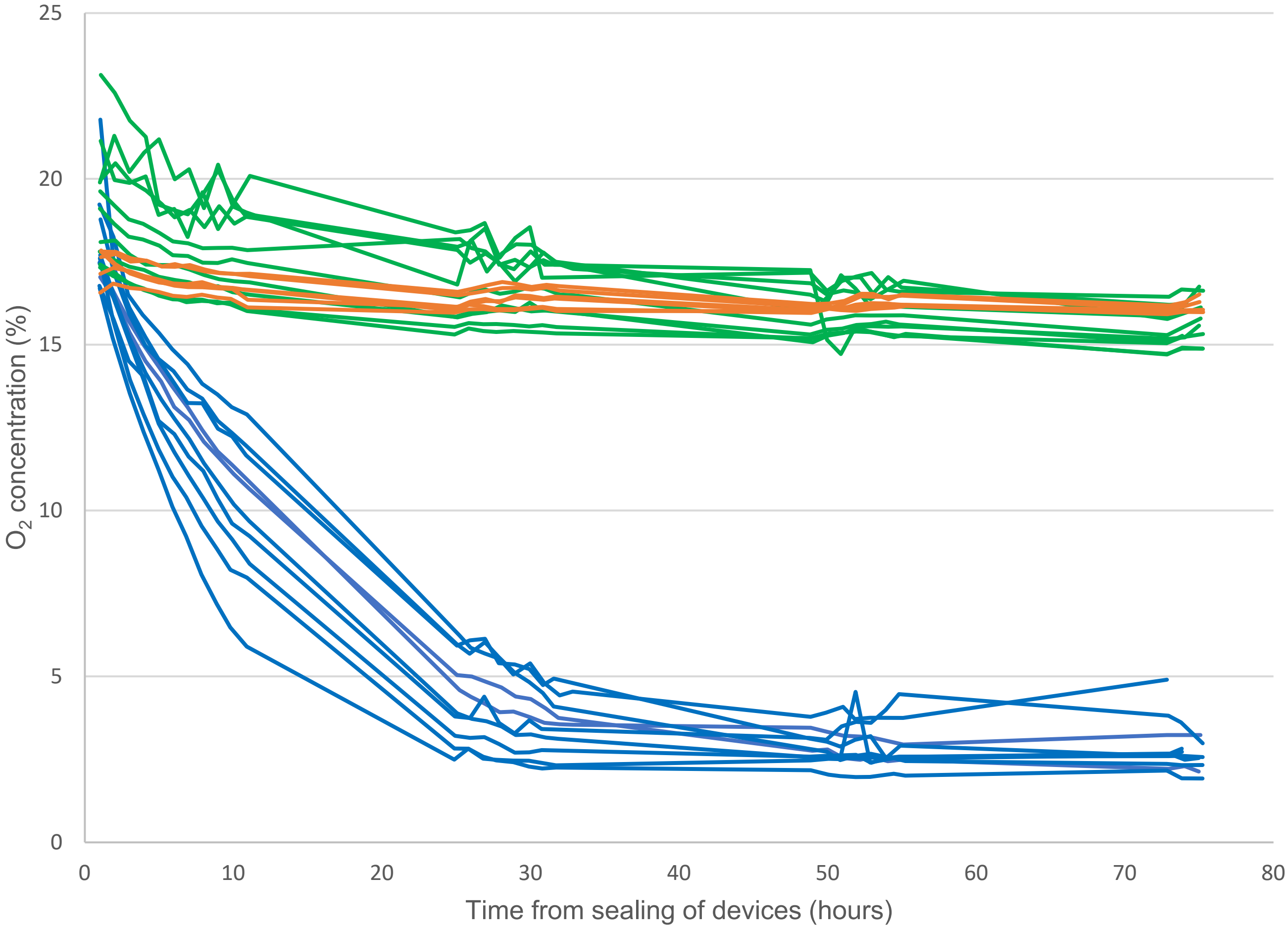

Figure S4: Raw data from monitoring of oxygen concentration. Results from monitoring the oxygen concentration during four days of culture of A549 cells in the three different device versions of the culture chamber module; sensor/glass (blue), sensor/PMP (green) and sensor/PDMS (orange) (see figure 2 and section 2.2.1 for details on device designs). The plot displays the raw data behind the plot in figure 7. Each line represents the oxygen measurements in one cell cultivation chamber.

S4 Oxygen supply to cells in culture

Delivery of oxygen to cells in culture

The demand of oxygen supply for cultured cells depends on the cells' oxygen consumption rate (OCR) (units: mol cell^{-1} s^{-1}) and number of cells per area in a cultivation device. Multiplying the two gives the oxygen consumption rate in units mol cm^{-2} s^{-1}.

In conventional cell culture the supply of oxygen happens by diffusion from the surrounding air through the cell culture medium and is given by Fick's first law:

$$F = D * \frac{\Delta C}{\Delta x} \tag{S1}$$

where F, D, C and x are the flux, diffusion coefficient, concentration and thickness of the medium layer. In the microfluidic culture chambers used in this work, the supply of oxygen happens by diffusion from the surrounding air through the PMP film and is given by Fick's first law for gas flux in a polymer film:

$$F = D * \frac{\Delta(p * S)}{\Delta x} = D * S * \frac{\Delta p}{\Delta x} = P * \frac{\Delta p}{\Delta x} \tag{S2}$$

Where p, S and P are the pressure, solubility coefficient and permeability of the relevant gas in the polymer. The unit of F is mol cm^{-2} s^{-1}.

Calculation of oxygen flux through PMP film and culture medium

Calculation of oxygen flux through culture medium for *in vitro* cell culture is thoroughly described by Place et al. [9]. The current calculations of flux through culture medium and PMP are performed accordingly. A schematic of the two set-ups is found in figure S5.

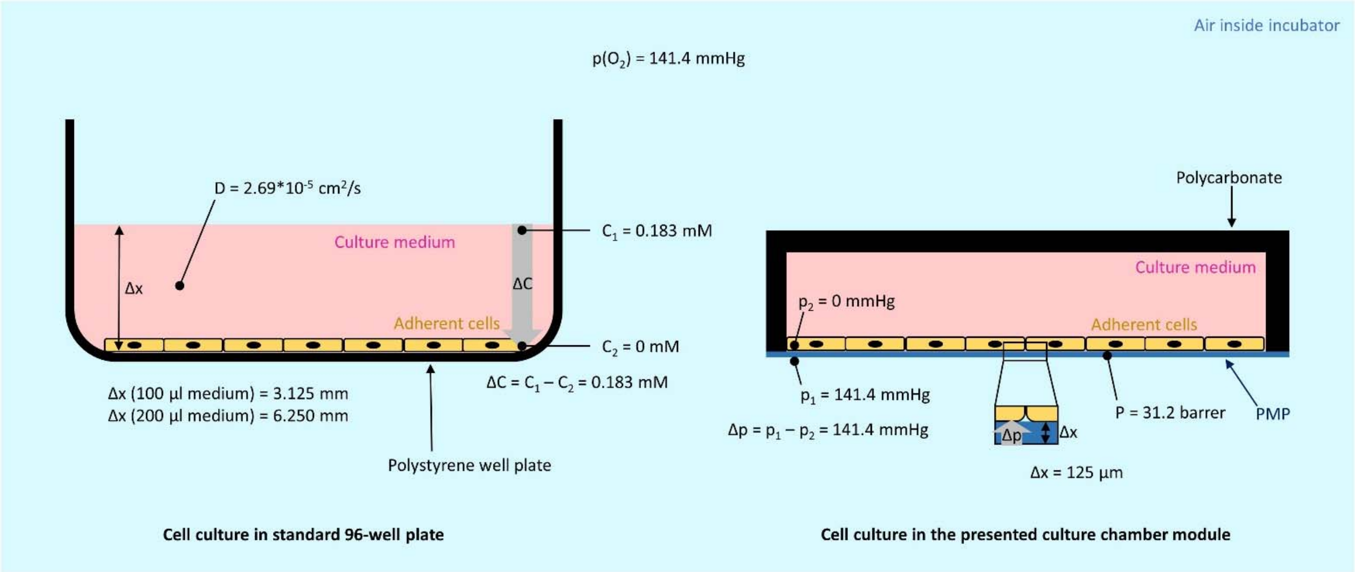

Figure S5: Schematic illustration with input parameters Schematic illustration of oxygen flux in standard cell culture in polystyrene well plates where oxygen diffuses through the culture medium, and oxygen flux in the presented culture chamber module where oxygen diffuses through the PMP material.

Input parameters Δp and ΔC

The cells are cultured in standard conditions, i.e., in a cell cultivation incubator at 37°C with 100% humidity and 5% CO_2. This reduces the gaseous oxygen concentration from 21% in dry air to 18.6% in the incubator [9]. At sea level with atmospheric pressure 1 atm = 760 mmHg, the partial pressure of oxygen is thus **141.4 mmHg** (760 mmHg * 18.6%) in the air inside the incubator.

At the interface between air and culture medium, the gaseous oxygen in the air is in equilibrium with dissolved oxygen in the medium. Henry's law describes the gas solubility in a liquid medium and states that the molar concentration of dissolved gas ($C_{(d)}$: mM) is directly proportional to the partial pressure of the gas ($P_{(g)}$: mmHg) of the overlying air and the Henry constant (H: mmHg/mM) [9].

$C_{(d)} = P_{(g)} / H$ Henry's law

Values for Henry's constant are difficult to estimate and need to be determined experimentally [9]. We utilize the value of H as given in Place et al. [9] applying for culture medium with an ionic strength of 175 mM (average for most mammalian culture mediums), with atmospheric pressure of 760 mmHg, at 37°C, which is H = 771.65 mmHg/mM. This gives a concentration of dissolved oxygen at the medium surface of

$C_{(d)}$ = 141.4 mmHg / 771.65 mmHg/mM = **0.183 mM**

We calculate the maximum flux of oxygen through the culture medium or PMP, hence for the situation where we set the partial pressure of oxygen / the concentration of dissolved oxygen at the cell layer to **0 mmHg / 0 mM**.

Consequently, the oxygen pressure difference across the PMP film, Δp, is

$\mathbf{\Delta p}$ = 141.4 mmHg – 0 mmHg = **141.4 mmHg**

And the oxygen concentration gradient from the medium surface to the cell layer, ΔC, is

$\mathbf{\Delta C}$ = 0.183 mM – 0 mM = **0.183 mM**

Input parameter Δx

For the situation of oxygen flux through the PMP film, Δx is the thickness of the film which is

$\mathbf{\Delta x_{PMP} = 125\ \mu m}$

For the situation of oxygen flux through culture medium, Δx is the thickness of the layer of medium and is thus calculated by

Δx = Volume / Surface area

For a standard 96-well plate, the recommended working volume is 100 – 200 µl and the surface area of the well is 32 mm^2 [40]. The thicknesses of the medium layers with the two volumes are thus

$\Delta x_{100\mu l}$ = 100 mm^3 / 32 mm^2 = 3.125 mm = **3125 µm**

$\Delta x_{200\mu l}$ = 200 mm^3 / 32 mm^2 = 6.250 mm = **6250 µm**

Input parameter P and D

The permeability of oxygen in the PMP film was measured in our experiments to be

P = 31.2 barrer

Where 1 barrer = 10^{-10} cm^3_{STP} * cm / (cm^2*s*cmHg))
1 cm^3_{STP} = (1 / 22414) mol = 4.46*10^{-5} mol

The diffusion coefficient of oxygen in culture medium is assumed to be

D = 2.69*10^{-5} cm^2/s

as reasoned for and utilized by Place et al. [9].

Calculated oxygen flux through PMP film and culture medium

The flux of oxygen through the PMP film:

$$F = P * \frac{\Delta p}{\Delta x}$$

$$F = 31.2 \text{ barrer} * \frac{141.4 \text{ mmHg}}{125 \text{ µm}}$$

$$F = 31.2 * 10^{-10} \text{ cm}^3(\text{STP}) * \text{ cm} / (\text{cm}^2 * \text{s} * \text{cmHg}) * \frac{14.14 \text{ cmHg}}{0.0125 \text{ cm}}$$

$$F = 3.529 * 10^{-6} \text{ cm}^3(\text{STP}) / (\text{cm}^2 * \text{s})$$

$$F = 1.575 * 10^{-10} \text{ mol} / (\text{cm}^2 * \text{s})$$

$$\boldsymbol{F = 157.5 \text{ pmol} / (\text{cm}^2 * \text{s})}$$

The flux of oxygen through 100 µl culture medium:

$$F = D * \frac{\Delta C}{\Delta x}$$

$$F = 2.69 * 10^{-5}\text{cm}^2/s * \frac{0.183\ \text{mM}}{3125\ \mu\text{m}}$$

$$F = 2.69 * 10^{-5}\text{cm}^2/s * \frac{0.183 * 10^{-6}\text{mol/cm}^3}{0.3125\ \text{cm}}$$

$$F = 1.575 * 10^{-11}\text{mol} / (\text{cm}^2 * \text{s})$$

$$\boldsymbol{F = 15.75\ \text{pmol} / (\text{cm}^2 * \text{s})}$$

The flux of oxygen through 200 µl culture medium:

$$F = D * \frac{\Delta C}{\Delta x}$$

$$F = 2.69 * 10^{-5}\text{cm}^2/s * \frac{0.183\ \text{mM}}{6250\ \mu\text{m}}$$

$$F = 2.69 * 10^{-5}\text{cm}^2/s * \frac{0.183 * 10^{-6}\text{mol/cm}^3}{0.6250\ \text{cm}}$$

$$F = 7.876 * 10^{-12}\text{mol} / (\text{cm}^2 * \text{s})$$

$$\boldsymbol{F = 7.876\ \text{pmol} / (\text{cm}^2 * \text{s})}$$

References

[9] T.L. Place, F.E. Domann, A.J. Case, Limitations of oxygen delivery to cells in culture: An underappreciated problem in basic and translational research, Free Radic. Biol. Med. 113 (2017) 311–322. https://doi.org/10.1016/J.FREERADBIOMED.2017.10.003.

[40] Useful Numbers for Cell Culture, (n.d.). https://www.thermofisher.com/no/en/home/references/gibco-cell-culture-basics/cell-culture-protocols/cell-culture-useful-numbers.html (accessed October 24, 2022).
